# Conceptual Design and Computational Modeling Analysis of a Single-Leg System of a Quadruped Bionic Horse Robot Driven by a Cam-Linkage Mechanism

**DOI:** 10.1155/2019/2161038

**Published:** 2019-11-04

**Authors:** Liangwen Wang, Weiwei Zhang, Caidong Wang, Fannian Meng, Wenliao Du, Tuanhui Wang

**Affiliations:** School of Mechanical and Electrical Engineering, Zhengzhou University of Light Industry, Henan Provincial Key Laboratory of Intelligent Manufacturing of Mechanical Equipment, Zhengzhou 450002, China

## Abstract

In this study, the configuration of a bionic horse robot for equine-assisted therapy is presented. A single-leg system with two degrees of freedom (DOFs) is driven by a cam-linkage mechanism, and it can adjust the span and height of the leg end-point trajectory. After a brief introduction on the quadruped bionic horse robot, the structure and working principle of a single-leg system are discussed in detail. Kinematic analysis of a single-leg system is conducted, and the relationships between the structural parameters and leg trajectory are obtained. On this basis, the pressure angle characteristics of the cam-linkage mechanism are studied, and the leg end-point trajectories of the robot are obtained for several inclination angles controlled by the rotation of the motor for the stride length adjusting. The closed-loop vector method is used for the kinematic analysis, and the motion analysis system is developed in MATLAB software. The motion analysis results are verified by a three-dimensional simulation model developed in Solidworks software. The presented research on the configuration, kinematic modeling, and pressure angle characteristics of the bionic horse robot lays the foundation for subsequent research on the practical application of the proposed bionic horse robot.

## 1. Introduction

The quadruped walking robots are an important type of legged robots. Recently, many countries have conducted profound research on the walking robots, which denote a frontier technology of the strategic significance. The BigDog developed by Boston Dynamics is a rough-terrain robot that captures the mobility, autonomy, and speed of living creatures, which is a typical example of a legged robot [1, 2]. The cheetah robots presented in [3] are capable of many functions, among which are sprinting and sharp turning, which is similar to the kinematics of the biological prototypes. The quadruped walking robots are equipped with multibranched motion mechanisms. The multi-degree-of-freedom coupling between the branches makes the coordination of robot motion very complicated [4]. In order to improve the mobility and load capacity of a robot, several researchers have extensively studied the structure, movement, and control of the robot legs. Li et al. [5] systematically studied a single-leg system of a quadruped bionic robot. Based on the analysis of the muscle-bone structure of quadrupeds, the DOF configuration of a single leg was determined. Chen et al. [6] studied a bionic quadruped robot in order to improve its dynamic stability and adaptability by imitating the quadrupeds. Chen et al. [7] designed a new bionic robot named Hound. The body structure, especially that of the legs, and geometric relationships of the Hound robot were designed based on the bionic research. Ananthanarayanan et al. [8] designed the bionic legs suitable for high-speed motion, taking into consideration the balance between weight and strength. Smith and Jivraj [9] compared a hind leg with a leg with a different arrangement, demonstrating in detail how leg orientation can affect the dynamic characteristics and gait performance of a robot. Seok et al. [10] introduced the design principles of the highly efficient walking robots, which were implemented in the design and experimental analysis of a cheetah robot.

Nowadays, it is relatively common to use robots in human rehabilitation and treatment processes. The robotized systems have been used for rehabilitation, improving the efficacy while reducing the healthcare costs [11]. The rehabilitation robots have significant advantages in helping patients to recover from neurological disorders and to become able to walk again [12]. Over the last few decades, many kinds of lower limb rehabilitation robots have been developed [13–15]; however, their high cost makes them impracticable for home healthcare.

Recently, several one-DOF mechanisms have been proposed for a rehabilitation process, such as the four-bar mechanisms proposed by Alves et al. [16], the Stephenson III six-bar mechanism [17], and the ten-bar linkage mechanism proposed by Tsuge and Mccarthy [18]. With the aim to overcome the limitation of such mechanisms that the desired gait path can be matched only approximately, Kay and Haws [19] proposed a cam-linkage mechanism, combining a fixed cam and a four-bar linkage to generate the desired path accurately. Mundo et al. [20] synthesized the cam-linkage mechanisms with one or more cams for precise path generation, while Soong [21] proposed a novel cam-geared mechanism for path generation. Shao et al. [22] designed a new robot structure, where a seven-bar crank-slider mechanism was combined with a cam to generate a precise target path. The novel lower-limb rehabilitation system was composed of a body weight support system to unload the body weight and two cam-linkage mechanisms to generate the natural gait trajectory and guide the feet of a patient.

As an unconventional therapy, an equine-assisted therapy provides horse riding training [23], which allows the user's pelvis and torso to feel the movement of the horse in order to improve balance control, promote trunk extension, make a rhythmic rotation of the trunk, and enhance endurance performance and cardiopulmonary function [24, 25]. At present, it is costly to use horses for equine-assisted therapy, so equestrian therapy is used as a relatively less-costly solution. In this study, in order to imitate the movement of a horse and achieve effective equine-assisted therapy, a bionic horse robot driven by a cam-linkage mechanism is designed.

Each leg of the proposed bionic horse robot has two driving motors, which can adjust the span and height of the leg motion path. In the normal walking by using the leg motion, only one driving motor is needed.

The designed cam-linkage mechanism uses a constant-breadth three-center cam. The constant-breadth cam mechanism belongs to the class of desmodromic or positive drive mechanisms. In the case of a parallel flat-faced double follower, the distance of contact points between the two flat-faced followers and cam denotes the cam breadth. The constant-breadth cams may be the circular-arc cams or having an arbitrary geometry and can operate either translating or oscillating a follower if the appropriate desmodromic conditions are established.

In the studies on the constant-breadth cam mechanisms, usually, the circular-arc cam profiles and displacement functions for the double-dwell follower were used, basically synthesized applying the cycloidal and polynomial motion curves to a monomial basis [26]. Rothbart [27] presented a constant-breadth circular-arc cam profile in which the follower movement denoted a double-dwell function. Qian [28] investigated a constant-diameter cam mechanism, which included a double roller follower with a planar movement that was joined to an output rocker arm, and used a graphical-analytical method to establish the relationship between the geometric parameters of the designed constant-diameter cam mechanism and the cam angles.

The circular-arc cams are easy to design, manufacture, and test, which makes them cheaper than others [29, 30]. Cardona et al. [31] studied two constant-breadth cam mechanisms and presented the equations for calculating the cam breadth when the translating follower is eccentric with an inclination, the radius of curvature of its profile and the sliding velocities of constant-breadth cam mechanisms with translating and oscillating followers.

In the proposed constant-breadth three-center cam design, a swing center point of the follower can slide along the guide way, resulting in a complex plane follower motion. Recently, there have been a few reports on the cam-linkage mechanism where a constant-breadth three-center cam was used [31].

From the aspects of the overall robot structure, the structure and the working principle of a single-leg system of the robot driven by a cam-linkage mechanism are discussed in detail. The kinematics modeling and analysis of the cam-linkage mechanism are carried out, and the relationships between the structural parameters and foot trajectory of a single leg are obtained. On this basis, the pressure angle characteristics of the cam-linkage mechanism are thoroughly investigated. The closed-loop vector method is used for the kinematic analysis, and MATLAB software is used for the development of the motion analysis system. Moreover, the trajectory cluster of a robot meeting the pressure angle conditions is obtained. The motion analysis results are verified by the simulation with a three-dimensional model established in Solidworks software.

The rest of the paper is organized as follows. In Section 2, the structure of the bionic horse robot driven by a cam-linkage mechanism is briefly introduced. The working principle of the proposed robot is explained in detail in Section 3. In Section 4, the proposed cam-linkage mechanism is presented and analyzed. In Section 5, the pressure angle characteristics of the proposed robot are studied. The calculation example, the motion simulation, and the obtained results are given in Section 6. Finally, the conclusions are drawn in Section 7.

## 2. Structure of a Bionic Horse Robot Driven by a Cam-Linkage Mechanism

**Figure 1:**
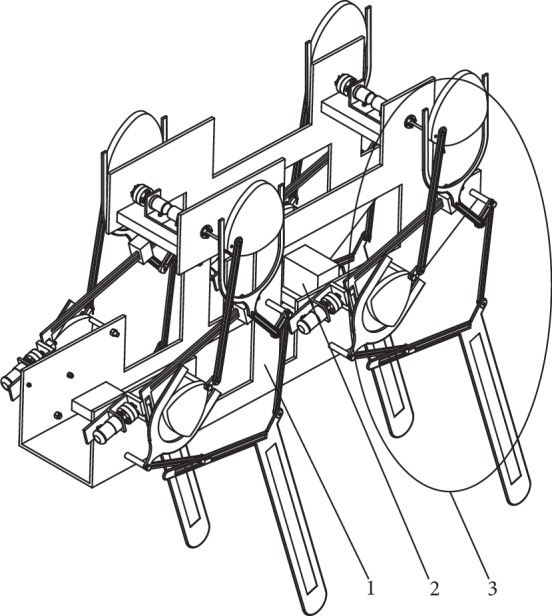
The structure of the bionic horse robot.

**Figure 2:**
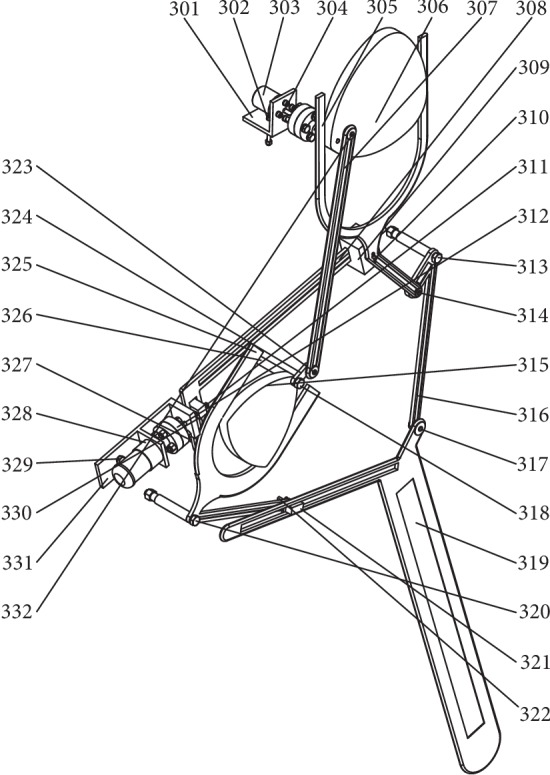
The structure of the single-leg walking system.

**Figure 3:**
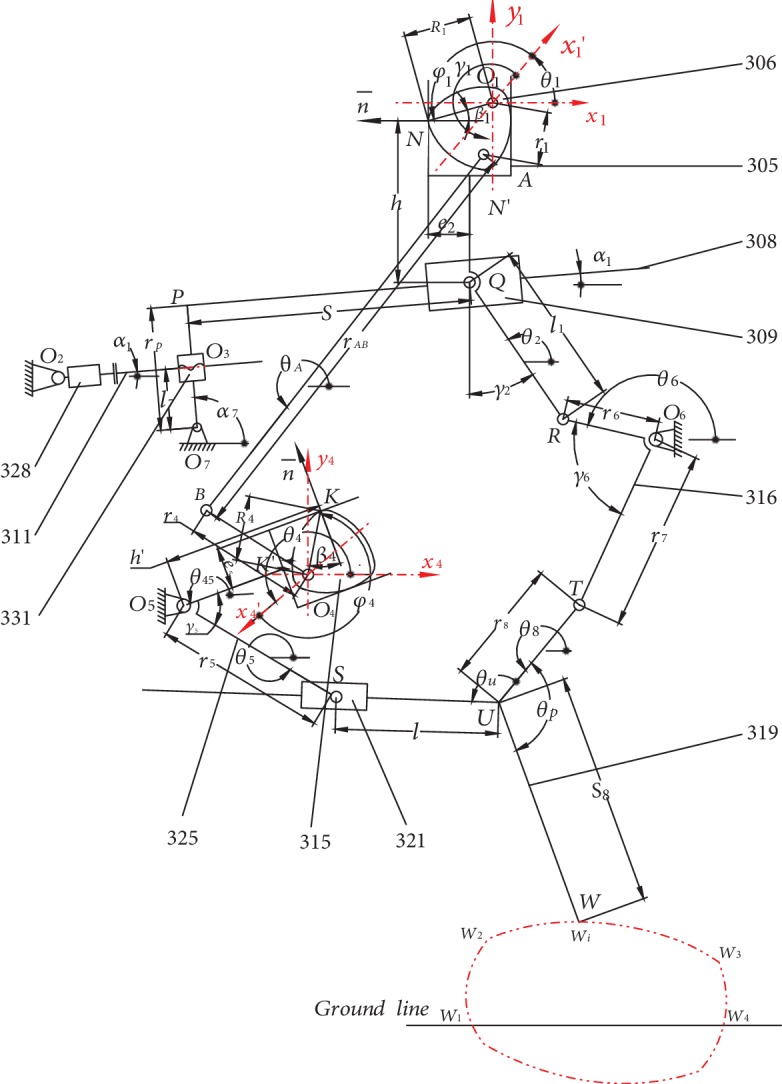
The schematic of the single-leg walking mechanism.

Following the bionic theory, a bionic horse robot imitates the movement of a horse. A single-leg motion of a bionic horse robot is driven by a cam-linkage mechanism. The overall structure of the robot is presented in Figure 1, where it can be seen that the robot consists of the robot body (denoted by 1), the control system (denoted by 2), and the single-leg walking system (denoted by 3). The developed robot had four parallel and symmetrical single-leg walking systems placed on both sides of the robot body. The structure of the single-leg walking system and the schematic of the single-leg walking mechanism are shown in Figures 2 and 3, respectively.

In Figures 2 and 3, 301 denotes the connection plate, 302 denotes the connection bolt, 303 denotes the driving motor for the stride length, 304 denotes the drive shaft of the stride length cam, 305 denotes the stride length fork, 306 denotes the stride length cam, 307 denotes the fork connecting rod, 308 denotes the rectangular swing rod, 309 denotes the short slide block, 310 denotes the connecting pin shaft I, 311 denotes the lead screw, 312 denotes the coupler, 313 denotes the body connecting pin shaft I, 314 denotes the connecting pin shaft II, 315 denotes the stride height cam, 316 denotes the connecting rod, 317 denotes the connecting pin shaft III, 318 denotes the connecting pin shaft IV, 319 denotes the walking leg, 320 denotes the body connecting pin shaft II, 321 denotes the long slider, 322 denotes the connecting pin shaft V, 323 denotes the connecting pin shaft VI, 324 denotes the short connecting rod, 325 denotes the stride height fork, 326 denotes the sleeve, 327 denotes the body connection pin shaft IV, 328 denotes the motor for the stride length adjusting, 329 denotes the body connection pin shaft V, 330 denotes the connection plate of the motor for the stride length adjusting, 331 denotes the nut slider, and lastly, 332 denotes the connection pin shaft VII.

The structure of the single-leg walking system includes the following mechanisms (see Figures 2 and 3):

1. The stride length mechanism, defined by the path *O*_1_*NN*′*QRO*_6_*O*_1_ in Figure 3, mainly determines the distance of the horizontal movement of the leg end-point *W*. During walking, driven by a driving motor for the stride length (303), astride length fork (305) moves and swings forward or backward along with the rotation of astride length cam. The motion of the stride length fork makes a connecting rod (316) to rotate, thus moving the walking leg (319) forward or backward
2. The stride length adjusting mechanism, defined by the path *O*_1_*O*_7_*O*_3_*O*_2_*O*_1_ in Figure 3, mainly controls the horizontal movement of the leg end-point *W*. A motor for the stride length adjusting (328) is connected with a lead screw (311) through a coupler. Rotation of the lead screw can change the inclination angle of a rectangular swing rod (308), which changes the motion locus of a stride length fork and controls the horizontal movement of the leg end-point *W*
3. The four-bar linkage mechanism, defined by the path *O*_1_*ABO*_4_*O*_1_ in Figure 3, mainly provides motion connection and transmission between the stride length cam (306) and the stride height cam (315), which transmits the motion of the driving motor for stride length to the lifting mechanism
4. The lifting mechanism, defined by the path *O*_1_*O*_4_*KK*′*O*_5_*O*_1_ in Figure 3, mainly determines the lifting distance of the leg end-point *W*. During the movement, a stride height cam (315) swings around the robot body. Driven by the stride height cam, a stride height fork (325) carries out a swinging motion. This motion causes the walking leg to lift through the long slider (321), controlling the lifting height of the motion path of the leg end-point *W*
5. The walking mechanism, defined by the path *O*_1_*O*_5_*SUTO*_6_*O*_1_ in Figure 3, represents a 2-DOF 5-bar linkage mechanism. Input motions denote two swings of the stride height fork (325) and the connecting rod (316), and lifting and forward/backward motions are formed, determining the motion path of the leg end-point *W*

## 3. Working Principle of a Bionic Horse Robot

When a bionic horse robot walks, all its four legs need to move simultaneously. The lifting height of a leg is mainly adjusted by the motor for the stride length adjusting. The driving motor for the stride length drives the stride length adjusting mechanism to produce the lifting and forward/backward motions of the leg.

The structure of the bionic horse robot is shown in Figure 1, and when the bionic horse robot moves, it firstly moves one leg forward while the other three legs are stationary supporting the robot body and keeping the robot stable. When that leg completes the lifting, stretching forward, and dropping actions, the leg end-point *W* touches the ground.

The motion path of the leg end-point *W*, shown in Figure 3, follows the trajectory from *W*_1_ to *W*_4_, passing through *W*_2_ and *W*_3_, where *W*_1_ is the starting point of the leg end-point before lifting from the ground and *W*_4_ is the contact point of the leg end-point after touching the ground. After the leg completes its movement, it remains stable to support the robot body, while the other three legs perform the same movement in turn. After all four legs have completed their movements and touched the ground, all four driving motors for stride length on the four legs drive their respective legs at the same time. At this point, the end-points of the four legs remain fixed on the ground, while pushing the robot body to move forward or backward. The stride length cam rotates for one cycle, and the robot moves forward or backward for one gait cycle. After one gait cycle is completed, each mechanism resets to its initial state and prepares for the next gait cycle.

Following Figures 2 and 3, and taking the leg forward movement as an example, we have the following. The driving motor for the stride length (303) drives the stride length cam (306), and the motion of the stride length cam is divided into two motion loops. (1) The four-bar mechanism (*O*_1_*ABO*_4_*O*_1_) causes the stride height cam to swing around the robot body. Further, the motion of the stride height cam causes the stride height fork to swing around the robot body. The motion of the stride height fork moves the long slider across the slide way of the leg. The motion of the long slider lifts the leg up and mainly controls the lifting portion in the motion path of the leg end-points. (2) The rotation of the stride length cam causes the motion of the stride length fork, and the motion of the stride length fork causes the connecting rod to swing around the robot body. The swinging of the connecting rod makes the leg move forward or backward. The stride length fork is attached to a short slide block that moves along the rectangular swing rod. Thus, by controlling the rotation of the motor for the stride length adjusting, the inclination angle of the rectangular swing rod can be adjusted.

**Figure 4:**
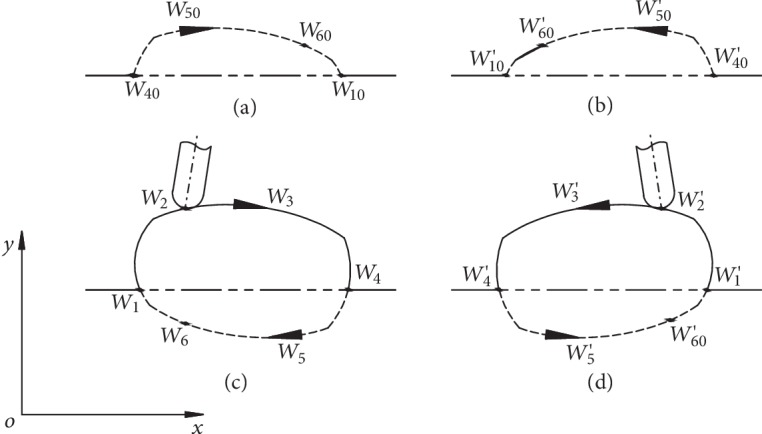
The gait of the bionic horse robot.

When the inclination angle of the rectangular swinging rod changes, the movement of the short slide block will cause the connecting rod to swing around the robot body at a different direction, which will result in the gait of the bionic horse robot shown in Figure 4. In Figure 4, (a) shows the movement trajectory of the robot body in a forward gait, (b) shows the movement trajectory of the robot body in a backward gait, (c) shows the movement trajectory of the leg end-point in a forward gait, and (d) shows the movement trajectory of the leg end-point in a backward gait.

As can be seen in Figure 4, when the leg is driven by the long slider and connecting rod, forming the leg end-point path from *W*_1_ to *W*_6_, passing through *W*_2_, *W*_3_, *W*_4_, and *W*_5_, successively (Figure 4(c)), a forward gait is generated. On the other hand, when the formed leg end-point path goes from *W*_1_′ to *W*_6_′, passing through *W*_2_′, *W*_3_′, *W*_4_′, and *W*_5_′, successively (Figure 4(d)), a backward gait is generated.

Here, a forward gait is used as an example to explain the robot movement principle. As already mentioned, when the robot begins to move, three legs support the body, while one leg moves following the trajectory defined by *W*_1_, *W*_2_, *W*_3_, and *W*_4_, successively. Afterwards, the other three walking legs perform the same movement one by one in a specific order, keeping the body relatively stable.

When all the four legs have moved from point *W*_1_ to point *W*_4_, then all of them are driven by the corresponding four driving motors for stride length simultaneously. At this point, the end-point of each leg *W* is fixed on the ground and pushes the robot body forward forming a robot body movement path (Figure 4(a)), where the mass center of the robot body moves along the trajectory *W*_40_-*W*_50_-*W*_60_-*W*_10_; this trajectory reveals an antisymmetric relationship with the theoretical trajectory of the leg end-point *W*_4_-*W*_5_-*W*_6_-*W*_1_, shown in Figure 4(c). In other words, while the leg is not moving from the end-point *W*, a full cycle movement is completed by the antisymmetric motion of the mass center of the robot body. After the robot body movement ends, the leg end-point *W*_4_ of the previous cycle becomes the starting point of the next cycle (new *W*_1_) and the robot body moves one step forward.

The trajectory of the leg end-point can be adjusted by the motor for stride length adjusting. Generally, the motor for stride length adjusting is locked in the process of a movement cycle, which means that it does not rotate. Namely, when the motor for stride length adjusting rotates, angle *α*_1_, defined by the horizontal part of the rectangular swing rod and the horizontal plane (see Figure 3), changes due to the motion of the nut slider. Any variation in *α*_1_ will change the motion range of the short slider on the rectangular swing rod; the motion of the short slider will affect the motion of the stride length fork, so that the swing angle of the connecting rod will change, altering the trajectory of the leg end-point and thus adjusting the stride length of the robot.

Usually, once the motor for stride length adjusting is set up, the trajectory of the leg end-point *W* is determined. The robot moves normally when the above movement is repeated continuously.

## 4. Kinematic Modeling and Analysis of a Bionic Horse Robot

A model for calculation of the trajectory of the leg end-point *W* is established based on the function of the stride length cam and the stride height cam.

### 4.1. Kinematic Relationships of a Constant-Breadth Three-Center Cam

**Figure 5:**
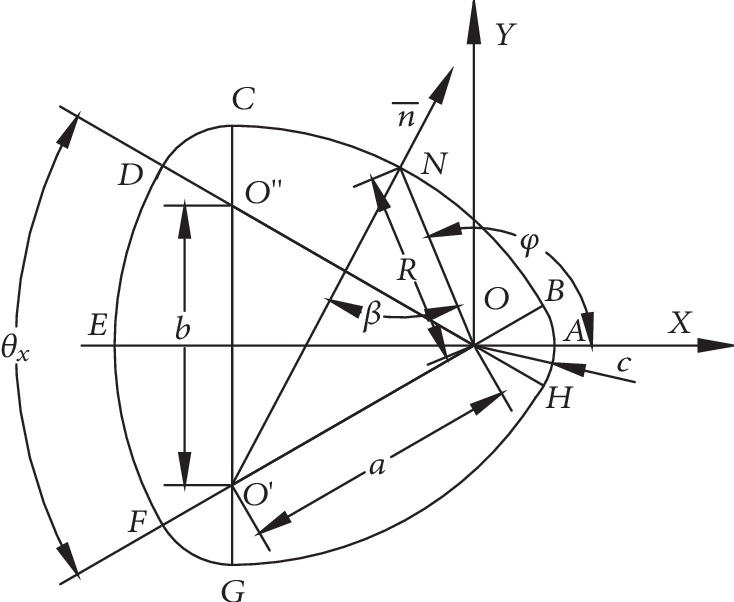
The constant-breadth three-center cam.

Both the stride length cam and the stride height cam of the bionic horse robot use the constant-breadth three-center cam, whose structure is shown in Figure 5. In Figure 5, it can be seen that the cam profile consists of six arc sections ($\overset{⌢}{HB}$, $\overset{⌢}{BC}$, $\overset{⌢}{CD}$, $\overset{⌢}{DF}$, $\overset{⌢}{FG}$, and $\overset{⌢}{GH}$). The centers of these arcs are *O*, *O*′, *O*^″^, *O*, *O*′, and *O*^″^, respectively.

The motion parameters of the constant-breadth three-center cam are given in Table 1. In Table 1, *a*, *b*, and *c* denote the structural parameters of the constant-breadth three-center cam, *O*, *O*′, and *O*^″^ are the rotation centers, *N* is the contact point between the cam and the follower, *φ* denotes the input angle, *β* denotes the output angle, and *R* denotes the radius vector.

In Table 1, it holds that

(1) $$\begin{array}{c} \theta_{x}=2\sin^{-1}\left(\frac{b}{2a}\right),\\ \text{SIGN}\varphi =\left\{\begin{array}{cc} 1, & \varphi \geq 0,\\ -1, & \varphi <0. \end{array}\right. \end{array}$$

### 4.2. Kinematic Analysis of a Stride Length Adjusting Mechanism

The closed loop of the stride length adjusting mechanism is given as *O*_1_*O*_7_*O*_3_*O*_2_*O*_1_ (see Figure 3). The kinematic relations of the stride length adjusting mechanism can be obtained by solving the following equation:

(2) XO7−XO2cosα1+YO7−YO2sinα=1l0±sθ32π,

where *l*_0_ is the initial length of the lead screw, *θ*_3_ is the rotation angle of the motor for stride length adjusting, and *α*_1_ is the angle between the lead screw and the horizontal direction. Using equation (2), a relationship between *α*_1_ and *θ*_3_ can be obtained.

It should be noted that in equation (2), symbol “+” stands for the positive rotation of the motor for stride length adjusting and symbol “-” stands for the reverse rotation of the motor for stride length adjusting.

### 4.3. Kinematic Analysis of a Stride Length Mechanism

The stride length mechanism is defined by *O*_1_*NN*′*QRO*_6_*O*_1_ (see Figure 3). Two closed loops, *O*_1_*NN*′*QPO*_7_*O*_1_ and *O*_1_*O*_7_*PQO*_6_*O*_1_, can be obtained by considering the stride length adjusting mechanism. From the closed loop *O*_1_*NN*′*QPO*_7_*O*_1_, we have

(3) $$\begin{array}{c} \vec{O_{1}N}+\vec{NN\prime }+\vec{N\prime Q}=\vec{O_{1}O_{7}}+\vec{O_{7}P}+\vec{PQ}, \end{array}$$

(4) R1ejθ1+φ1+hejθ1+φ+1β10+π/2+e2ejθ1+φ+1β10+π=XO7+YO7j+rpejα1+π/2+Sejα1,

(5) R1cosθ1+φ1+hcosθ1+φ+1β10+π2+e2cosθ1+φ+1β10+π=XO7+rpcosα1+π2+Scosα1,R1sinθ1+φ1+hsinθ1+φ+1β10+π2+e2sinθ1+φ+1β10+π=YO7+rpsinα1+π2+Ssinα1.

Further, from the closed loop *O*_1_*O*_7_*PQO*_6_*O*_1_, we have

(6) $$\begin{array}{c} \vec{O_{1}O_{7}}+\vec{O_{7}P}+\vec{PQ}+\vec{QR}=\vec{O_{1}O_{6}}+\vec{O_{6}R}, \end{array}$$

(7) $$\begin{array}{c} X_{O_{7}}+Y_{O_{7}}j+r_{p}e^{j\left(\alpha_{1}+\pi /2\right)}+{Se}^{j\alpha_{1}}+l_{1}e^{\theta_{2}}=X_{O_{6}}+Y_{O_{6}}j+r_{6}e^{j\theta_{6}}, \end{array}$$

(8) $$\begin{array}{c} \left\{\begin{array}{c} X_{O_{7}}+r_{p}\cos\left(\alpha_{1}+\frac{\pi }{2}\right)+S\cos\alpha_{1}+l_{1}\cos\theta_{2}=X_{O_{6}}+r_{6}\cos\theta_{6},\\ Y_{O_{7}}+r_{p}\sin\left(\alpha_{1}+\frac{\pi }{2}\right)+S\sin\alpha_{1}+l_{1}\sin\theta_{2}=Y_{O_{6}}+r_{6}\sin\theta_{6}, \end{array}\right. \end{array}$$

where *β*_10_ = −*β*_1_SIGN(*φ*_1_) and *θ*_2_ = *π*/2 + *θ*_1_ + *β*_10_ + *φ*_1_ + *γ*_2_, and *θ*_1_ denotes the input angle of the driving motor for the stride length. When structural parameters *a*_1_, *b*_1_, and *c*_1_ of the stride length cam are determined, the values of *φ*_1_, *h*, *S*, and *θ*_6_ can be obtained by combining the relations given in Table 1 with equations (5) and (8) and using the Newton iteration method.

### 4.4. Kinematic Analysis of a Four-Bar Linkage

From the closed loop *O*_1_*ABO*_4_*O*_1_ (see Figure 3), we have

(9) $$\begin{array}{c} \vec{O_{1}A}+\vec{AB}=\vec{O_{1}O_{4}}+\vec{O_{4}B}, \end{array}$$

(10) $$\begin{array}{c} r_{1}e^{j\left(\theta_{1}+\gamma_{1}\right)}+r_{AB}e^{j\theta_{A}}=X_{O_{4}}+Y_{O_{4}}j+r_{4}e^{j\left(\theta_{4}+\gamma_{4}\right)}, \end{array}$$

(11) $$\begin{array}{c} \left\{\begin{array}{c} r_{1}\cos\left(\theta_{1}+\gamma_{1}\right)+r_{AB}\cos\theta_{A}=X_{O_{4}}+r_{4}\cos\left(\theta_{4}+\gamma_{4}\right),\\ r_{1}\sin\left(\theta_{1}+\gamma_{1}\right)+r_{AB}\sin\theta_{A}=Y_{O_{4}}+r_{4}\sin\left(\theta_{4}+\gamma_{4}\right), \end{array}\right. \end{array}$$

where *θ*_*A*_ and *θ*_4_ can be obtained from equation (11) by using the Newton iteration method.

### 4.5. Motion Analysis of a Lifting Mechanism

The closed loop of the lifting mechanism is *O*_1_*O*_4_*KK*′*O*_5_*O*_1_ (see Figure 3), and based on it, the following equations can be obtained:

(12) $$\begin{array}{c} \vec{O_{1}O_{4}}+\vec{O_{4}K}+\vec{KK\prime }+\vec{K\prime O_{5}}=\vec{O_{1}O_{5}}, \end{array}$$

(13) $$\begin{array}{c} X_{O_{4}}+Y_{O_{4}}j+R_{4}e^{j\left(\theta_{4}+\varphi_{4}\right)}+h^{\prime }e^{j\left(\theta_{4}+\varphi_{4}+\beta_{40}+\pi /2\right)}+e_{5}e^{j\left(\theta_{4}+\varphi_{4}+\beta_{40}+\pi \right)}=X_{O_{5}}+Y_{O_{5}}j, \end{array}$$

(14) $$\begin{array}{c} \left\{\begin{array}{c} X_{O_{4}}+R_{4}\cos\left(\theta_{4}+\varphi_{4}\right)+h^{\prime }\cos\left(\theta_{4}+\varphi_{4}+\beta_{40}+\frac{\pi }{2}\right)+e_{5}\cos\left(\theta_{4}+\varphi_{4}+\beta_{40}+\pi \right)=X_{O_{5}},\\ Y_{O_{4}}+R_{4}\sin\left(\theta_{4}+\varphi_{4}\right)+h^{\prime }\sin\left(\theta_{4}+\varphi_{4}+\beta_{40}+\frac{\pi }{2}\right)+e_{5}\sin\left(\theta_{4}+\varphi_{4}+\beta_{40}+\pi \right)=Y_{O_{5}}, \end{array}\right. \end{array}$$

where *β*_40_ = −*β*_4_SIGN(*φ*_4_), and *φ*_4_ and *h*′ can be obtained from equation (14) by using the Newton iteration method.

### 4.6. Motion Analysis of a Leg Mechanism

The closed loop of the leg mechanism is *O*_1_*O*_5_*SUTO*_6_*O*_1_ (see Figure 3). From this closed loop, we have

(15) $$\begin{array}{c} \vec{O_{1}O_{5}}+\vec{O_{5}S}=\vec{O_{1}O_{6}}+\vec{O_{6}T}+\vec{TU}+\vec{US}, \end{array}$$

(16) $$\begin{array}{c} X_{O_{5}}+Y_{O_{5}}j+r_{5}e^{j\theta_{5}}=X_{O_{6}}+Y_{O_{6}}j+r_{7}e^{j\left(\theta_{6}+\gamma_{6}\right)}+r_{8}e^{j\theta_{8}}+{le}^{j\left(\theta_{U}+\theta_{8}-\pi \right)}, \end{array}$$

(17) $$\begin{array}{c} \left\{\begin{array}{c} X_{O_{5}}+r_{5}\cos\theta_{5}=X_{O_{6}}+r_{7}\cos\left(\theta_{6}+\gamma_{6}\right)+r_{8}\cos\theta_{8}+l\cos\left(\theta_{U}+\theta_{8}-\pi \right),\\ Y_{O_{5}}+r_{5}\sin\theta_{5}=Y_{O_{6}}+r_{7}\sin\left(\theta_{6}+\gamma_{6}\right)+r_{8}\sin\theta_{8}+l\sin\left(\theta_{U}+\theta_{8}-\pi \right), \end{array}\right. \end{array}$$

where *θ*_5_ = 3*π*/2 + *θ*_4_ + *β*_40_ + *φ*_4_ − *γ*_5_, and *θ*_8_ and *l* can be obtained from equation (17) also by using the Newton iteration method.

### 4.7. Trajectory Coordinate of Leg End-Point

Following the schematic of the single-leg walking mechanism presented in Figure 3, and solving equations (5), (8), (11), (14), and (17), the parameters *l*_7_, *α*_1_, *θ*_6_, *h*, *S*, *θ*_5_, and *θ*_8_ can be calculated, and the trajectory coordinates of the leg end-point can be obtained by

(18) $$\begin{array}{c} \left\{\begin{array}{c} X_{W}=X_{O_{6}}+r_{7}\cos\left(\theta_{6}+\gamma_{6}\right)+r_{8}\cos\theta_{8}+S_{8}\cos\left(\pi +\theta_{8}-\theta_{P}\right),\\ Y_{W}=Y_{O_{6}}+r_{7}\sin\left(\theta_{6}+\gamma_{6}\right)+r_{8}\sin\theta_{8}+S_{8}\sin\left(\pi +\theta_{8}-\theta_{P}\right), \end{array}\right. \end{array}$$

where *X*_*W*_ and *Y*_*W*_ are the trajectory coordinates of the leg end-point *W* in the horizontal and vertical directions, respectively.

## 5. Analysis and Discussion of Pressure Angle Performance

The pressure angle refers to a sharp angle formed between the normal on the cam profile on the contact point and the velocity direction of the corresponding contact point of the follower. In the following, the pressure angle of the stride length cam in the cam-linkage mechanism is discussed.

**Figure 6:**
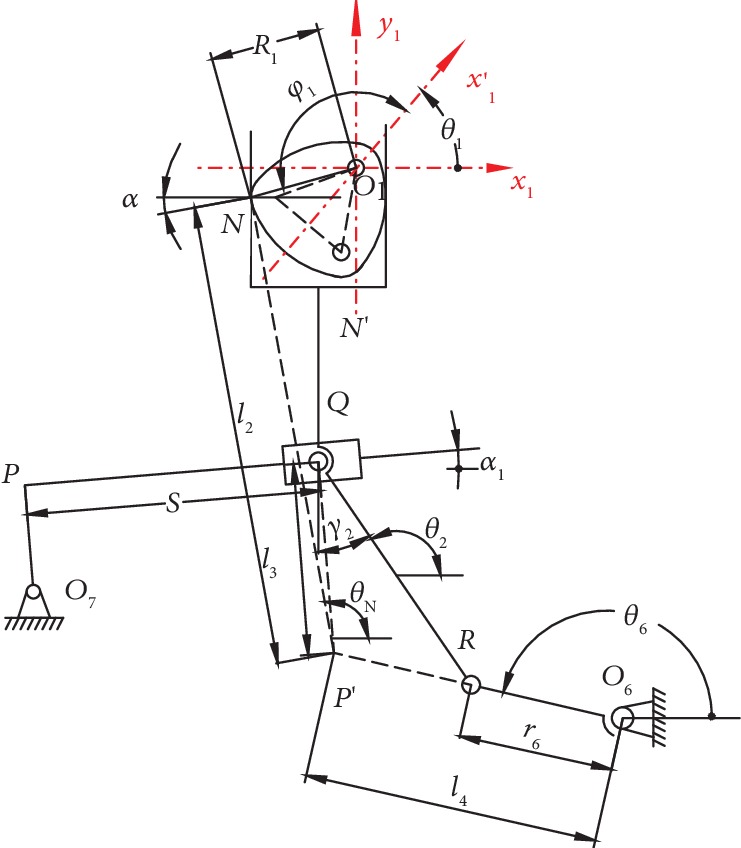
Calculation model of the pressure angle of the stride length cam.

The calculation model of the pressure angle of the stride length cam is illustrated in Figure 6. According to the three-center theorem of the velocity instantaneous center, the three velocity instantaneous centers of three adjacent components must be on the same straight line. If the velocity instantaneous centers of two adjacent component pairs are determined, the velocity instantaneous center of another pair of components can be obtained based on the mentioned theorem. It can be determined that point *P*′ is the velocity instantaneous center of the stride length fork that touches the stride length cam (Figure 6).

The pressure angle *α* can be calculated by setting up the loop equations. From the closed loop *O*_7_*PQP*′*O*_6_*O*_7_, we have

(19) $$\begin{array}{c} \vec{O_{7}O_{6}}+\vec{O_{6}P\prime }=\vec{O_{7}P}+\vec{PQ}+\vec{QP\prime }, \end{array}$$

(20) $$\begin{array}{c} \left(X_{O_{6}}-X_{O_{7}}\right)+\left(Y_{O_{6}}-Y_{O_{7}}\right)j+l_{4}e^{j\theta_{6}}=r_{P}e^{j\left(\alpha_{1}+\pi /2\right)}+{Se}^{j\left(\alpha_{1}+\pi \right)}+l_{3}e^{j\left(\alpha_{1}+3\pi /2\right)}, \end{array}$$

(21) $$\begin{array}{c} \left\{\begin{array}{c} \left(X_{O_{6}}-X_{O_{7}}\right)+l_{4}\cos\theta_{6}=r_{P}\cos\left(\alpha_{1}+\frac{\pi }{2}\right)+S\cos\left(\alpha_{1}+\pi \right)+l_{3}\cos\left(\alpha_{1}+\frac{3\pi }{2}\right),\\ \left(Y_{O_{6}}-Y_{O_{7}}\right)+l_{4}\sin\theta_{6}=r_{P}\sin\left(\alpha_{1}+\frac{\pi }{2}\right)+S\sin\left(\alpha_{1}+\pi \right)+l_{3}\sin\left(\alpha_{1}+\frac{3\pi }{2}\right). \end{array}\right. \end{array}$$

From the closed loop *O*_1_*O*_6_*P*′*NO*_1_, we have

(22) $$\begin{array}{c} \vec{O_{1}O_{6}}+\vec{O_{6}P\prime }+\vec{P\prime N}=\vec{O_{1}N}, \end{array}$$

(23) $$\begin{array}{c} X_{O_{6}}+Y_{O_{6}}j+l_{4}e^{j\theta_{6}}+l_{2}e^{j\theta_{N}}=R_{1}e^{j\left(\theta_{1}+\varphi_{1}\right)}, \end{array}$$

(24) $$\begin{array}{c} \left\{\begin{array}{c} X_{O_{6}}+l_{4}\cos\theta_{6}+l_{2}\cos\theta_{N}=R_{1}\cos\left(\theta_{1}+\varphi_{1}\right),\\ Y_{O_{6}}+l_{4}\sin\theta_{6}+l_{2}\sin\theta_{N}=R_{1}\sin\left(\theta_{1}+\varphi_{1}\right). \end{array}\right. \end{array}$$

By solving equations (21) and (24), *l*_2_, *l*_3_, *l*_4_, and *θ*_*N*_ can be obtained. Then, it holds that

(25) $$\begin{array}{c} \alpha =\left(\theta_{N}+\frac{\pi }{2}\right)-\left(\theta_{1}+\varphi_{1}+\beta_{10}\right), \end{array}$$

where *β*_10_ = −*β*_1_SIGN(*φ*_1_).

## 6. Calculation Example and Simulation

In this section, a calculation example and a motion simulation are given. The structural parameters of the designed single-leg system were as follows. Note that all units are given in mm. The center *O*_1_ of the stride length cam was used as the origin of the coordinate system (see Figure 3), and the coordinates of the points were as follows: *O*_7_ (-360, -753), *O*_2_ (-460, -720), *O*_6_ (383, -531), *O*_4_ (-114, -654), and *O*_5_ (-433, -923). The lengths where as follows: *r*_*p*_ = 185(length of *O*_7_*P*), *l*_1_ = 240 (length of *QR*), *e*_2_ = 165 (single-side width of the stride length fork), *r*_4_ = 44 (length of *BO*_4_), *r*_1_ = 35 (length of *O*_1_*A*), *r*_*AB*_ = 655 (length of *AB*), *r*_7_ = 474 (length of *O*_6_*T*), *r*_6_ = 95 (length of *RO*_6_), *r*_8_ = 100 (length of *TU*), *S*_8_ = 1081 (distance between point *U* and point *W*), *r*_5_ = 356 (length of *O*_5_*S*), and *e*_5_ = 120 (single-side width of the stride height fork). Additionally, the angles were *γ*_1_ = 180°, *γ*_2_ = 40°, *γ*_5_ = 62°, *γ*_6_ = 40°, *γ*_4_ = −8°, *θ*_*U*_ = 120°, and *θ*_*P*_ = 125°. The structural parameters of the stride cam were *a*_1_ = 220, *b*_1_ = 160, and *c*_1_ = 25, while those of the stride height cam were *a*_2_ = 220, *b*_2_ = 220, and *c*_2_ = 10. MATLAB software was used for the development of the motion analysis system for the bionic horse.

### 6.1. Calculation Example

**Figure 7:**
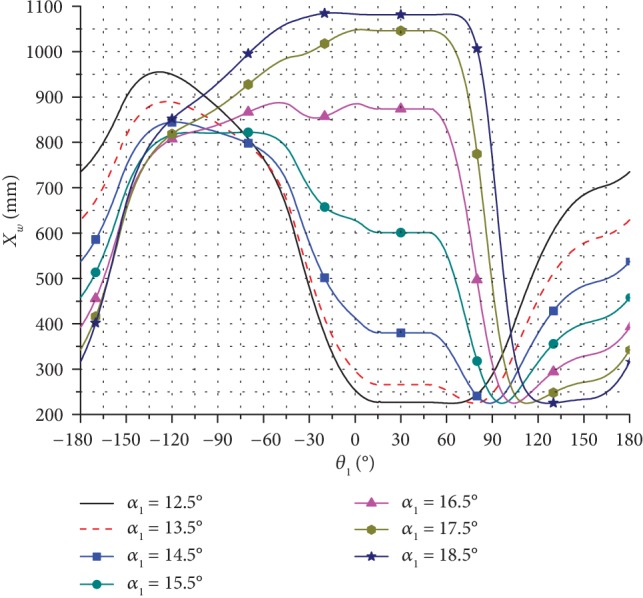
The horizontal direction trajectories of the leg end-point for moving forward at different inclination angles.

**Figure 8:**
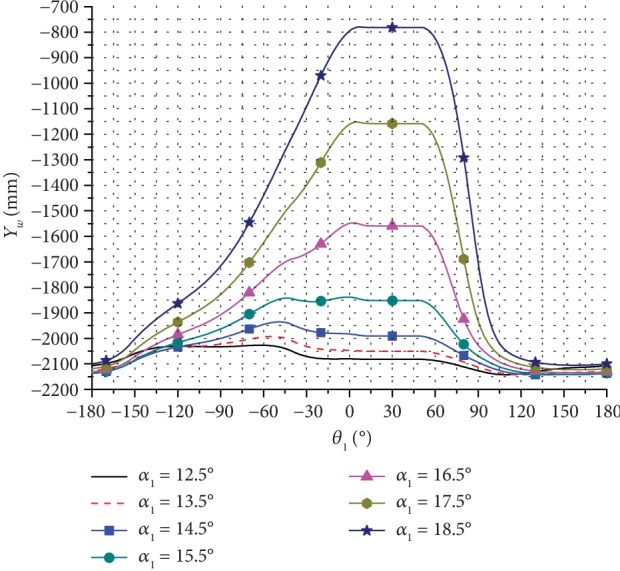
The vertical direction trajectories of the leg end-point for moving forward at different inclination angles.

**Figure 9:**
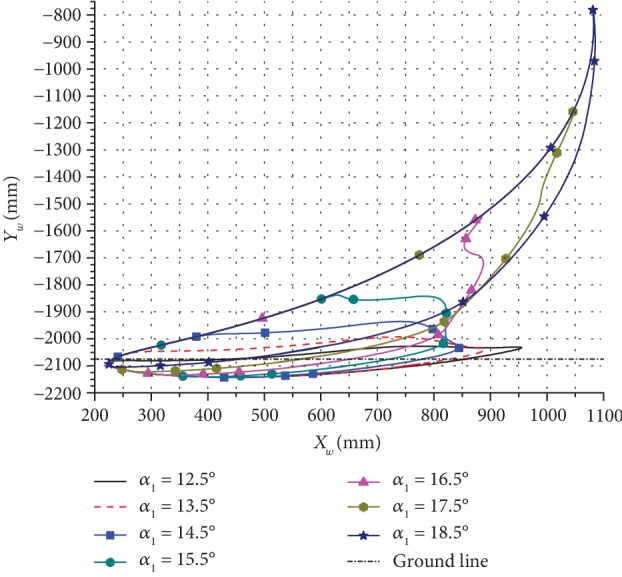
The trajectories of the leg end-point for moving forward at different inclination angles.

Based on the kinematic model of the bionic horse robot and the related input parameters, the change in the motion trajectory and the pressure angle for one cycle was calculated. Several trajectories of the leg end-point were obtained by changing the inclination angle *α*_1_ of the rectangular swing rod, and the obtained results are presented in Figures 7–9.

As can be seen in Figure 9, when *α*_1_ increased, the height of the leg end-point trajectory changed significantly.

**Figure 10:**
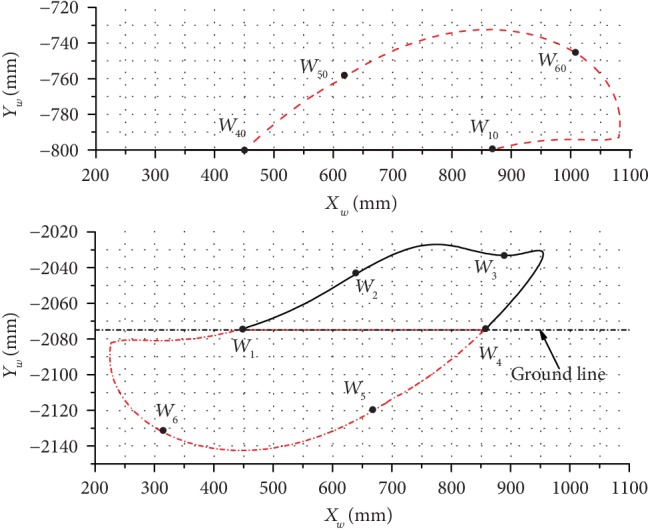
The trajectories of the leg end-point (bottom) and the body (top) at *α*_1_ = 12.5°.

**Figure 11:**
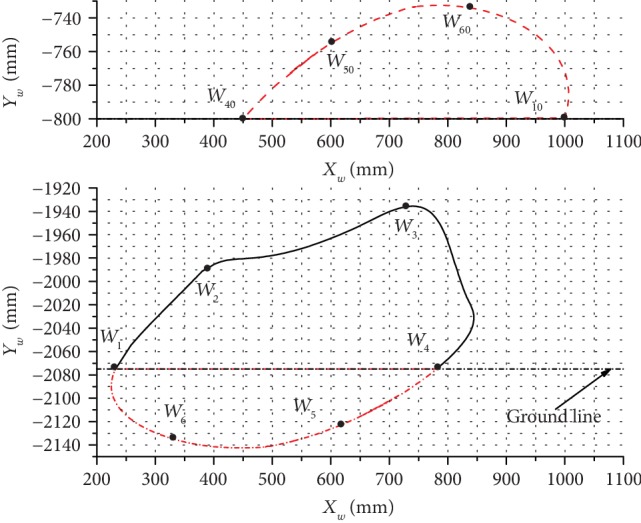
The trajectories of the leg end-point (bottom) and the body (top) at *α*_1_ = 14.5°.

**Figure 12:**
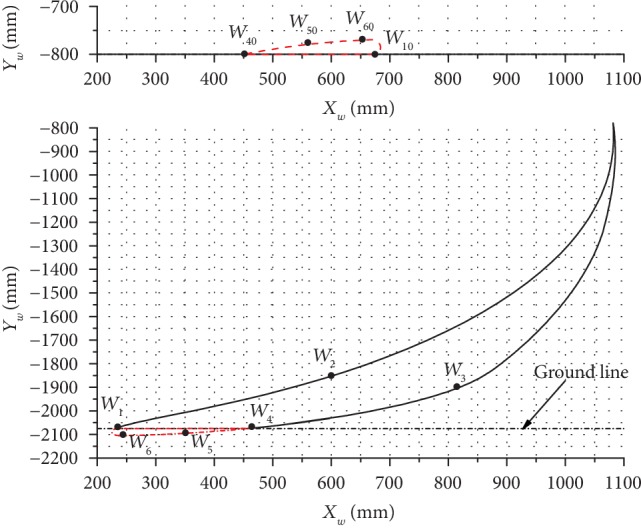
The trajectories of the leg end-point (bottom) and the body (top) at *α*_1_ = 18.5°.

The inclination angles of 12.5°, 14.5°, and 18.5° were used to analyze and explain the changes in the leg end-point trajectory and the body trajectory, and analysis results are presented in Figures 10–12.

As can be seen in Figure 10, at the inclination angle of 12.5°, the maximum lifting height of the leg end-point *W* was 47.72 mm and the maximum horizontal moving distance was 506.33 mm. For the robot body, the maximum change in height was 67.76 mm, the maximum horizontal moving distance was 631.22 mm, and the stride length of this kind of gait was 417.18 mm.

The results obtained at the inclination angle of 14.5° are presented in Figure 11, where it can be seen that the maximum lifting height of the leg end-point *W* was 139.68 mm and the maximum horizontal moving distance was 611.79 mm. For the robot body, the maximum change in height was 67.13 mm, the maximum horizontal moving distance was 555.32 mm, and the stride length of this kind of gait was 548.59 mm.

Lastly, the results obtained at the inclination angle of 18.5° are shown in Figure 12, where it can be seen that the maximum lifting height of *W* was 1293.14 mm and the maximum horizontal moving distance was 852.04 mm. For the robot body, the maximum change in height was 30.62 mm, the maximum horizontal moving distance was 232.85 mm, and the stride length of this kind of gait was 226.83 mm. Based on the obtained results it can be concluded that when this kind of leg end-point trajectory (shown in Figure 12) is used for the gait of the bionic horse robot, a large lifting height of the leg can be achieved, but the moving distance of the body will be small, which is similar to the horse strolling in situ.

Generally speaking, at *α*_1_ = 14.5°, this leg end-point trajectory (shown in Figure 11) for the gait of the bionic horse robot would be a better choice.

**Figure 13:**
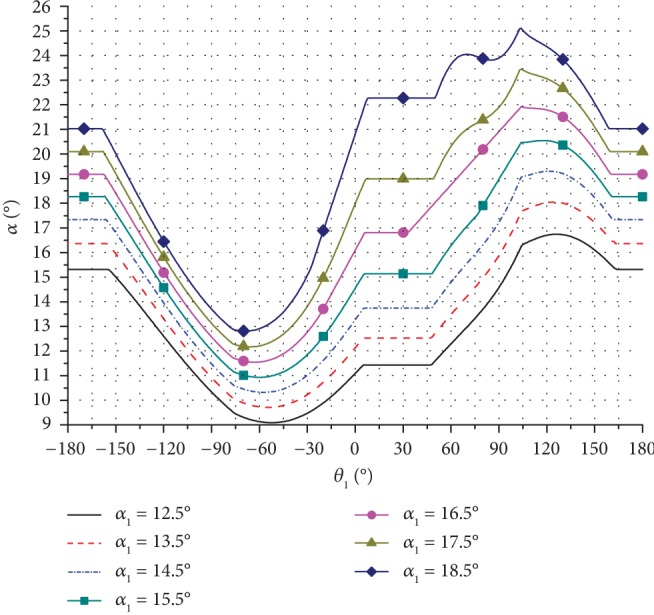
Pressure angles of the stride length cam mechanism during the forward gait for different inclination angles.

The pressure angles of the stride length cam mechanism during the forward gait were also determined, and the obtained values are presented graphically in Figure 13. In Figure 13, the rotation angle of the stride length cam is shown on the horizontal axis and the inclination angle is presented on the vertical axis; different line types are used to show the pressure angle for different values of the inclination angle.

In general, the maximum allowed pressure angle was 45°. Also, as can be seen in Figure 13, the maximum angle of the stride length cam mechanism was less than 26°, which was within the allowed range.

### 6.2. Motion Simulation

**Figure 14:**
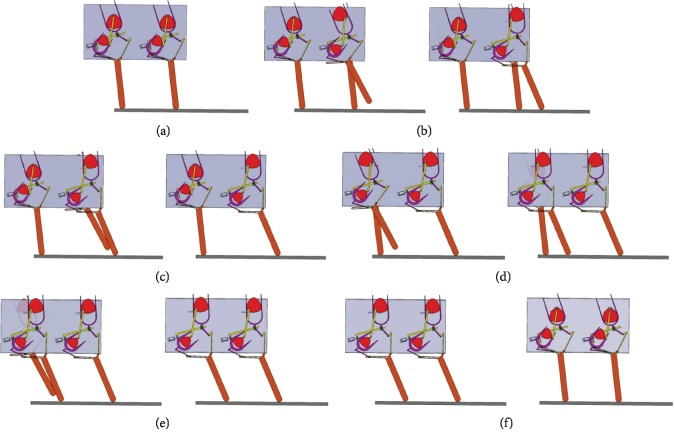
A forward motion cycle simulation for the robot entirety at *α*_1_ = 14.5° in Solidworks software.

**Figure 15:**
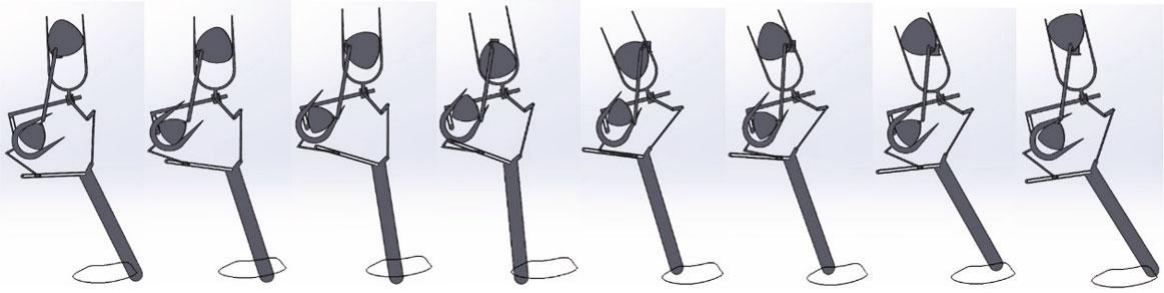
The forward leg motion simulation at *α*_1_ = 14.5° in Solidworks software.

In order to validate the calculated values of the motion trajectory and pressure angle of the bionic horse, a structural model of the robot was developed, and its motion was simulated in Solidworks software. The model of the forward leg motion at *α*_1_ = 14.5° of the bionic horse robot in the simulation software is presented in Figures 14 and 15.

A forward motion cycle simulation for the robot entirety is shown in Figure 14, (a) shows the initial position of the robot, (b) shows the forward movement of the first leg, (c) shows the forward movement of the second leg, (d) shows the forward movement of the third leg, (e) shows the forward movement of the fourth leg, and (f) shows the movement of the robot body completing a forward gait.

The forward motion simulation for one leg is shown in Figure 15. The motion trajectory of the leg and the pressure angle of the cam were automatically exported by the simulation software.

**Figure 16:**
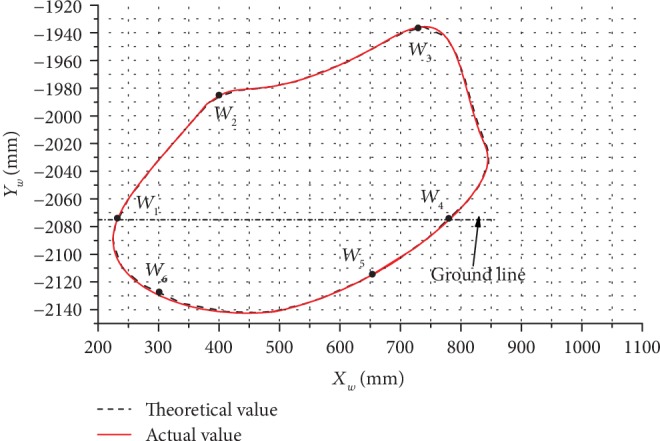
The trajectory of the leg end-point during the forward gait at *α*_1_ = 14.5°.

In Figure 16, the trajectory of the leg end-point *W* during the forward gait at *α*_1_ = 14.5° obtained by both the motion simulation (solid line) and the theoretical calculation (dotted line) is shown. As can be seen in Figure 16, the maximum differences between the motion simulation trajectory and theoretically calculated trajectory were 4.60 mm in the *x*-direction and 2.15 mm in the *y*-direction.

**Figure 17:**
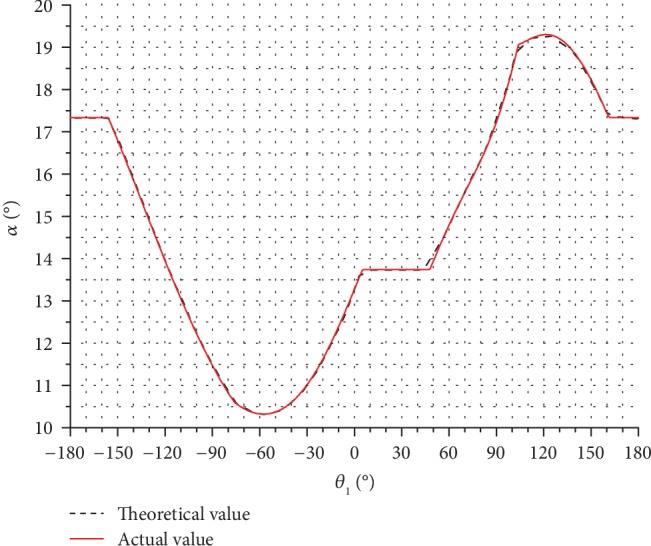
The pressure angle of the stride length cam during the forward gait at *α*_1_ = 14.5°.

In Figure 17, the pressure angle curve of the stride length cam during the forward gait at *α*_1_ = 14.5° obtained by both the motion simulation (solid line) and the theoretical calculation (dotted line) is shown. The maximum differences in the pressure angle between the motion simulation and the theoretical calculation were 0.21°.

Based on the results demonstrated in Figures 16 and 17, the differences in results between the motion simulation and the theoretical calculation were minimal; thus, the motion trajectory and pressure angle calculation models were verified.

## 7. Conclusion

In this study, a quadruped bionic horse robot driven by a cam-linkage mechanism is proposed. It is demonstrated that by using the cam-linkage mechanism in a single-leg system, the control system of a robot can be significantly simplified. Also, it is shown that the proposed bionic horse robot can follow several different body motion trajectories, which is very significant for equine-assisted therapy. Moreover, both modeling and analysis are conducted to obtain the motion trajectory of the leg end-point and pressure angle curve of the stride length cam for the leg of the bionic horse robot. The results presented in this work lay the foundation for the functionalization and structural optimization of the bionic horse robot driven by a cam-linkage mechanism.

For the proposed quadruped bionic horse robot driven by a cam-linkage mechanism, in terms of the adaptability of a walk floor and the diversity of the motion gaits, it is likely to be weaker than a typical multilegged walking robot, whose walking legs are composed of a series of multi-DOF linkages. However, the equestrian therapy is usually performed on a flat floor, which requires a relatively simple change in gait. A malfunction of the leg is less likely because of out of plane forces. The cost of manufacturing and using the quadruped bionic horse robot proposed is a relatively less-costly solution, which may be a good selection for equestrian therapy.

## Figures and Tables

**Table 1:** Motion parameters of the constant-breadth three-center cam.

Absolute value of input angle |*φ*|	Output angle *β*	Radius vector *R*
$\left|\varphi \right|\leq \sin^{-1}\frac{b}{2a}$	0	*c*
$\sin^{-1}\frac{b}{2a}<\left|\varphi \right|\leq \frac{\pi }{2}+\tan^{-1}\frac{\sqrt{a^{2}-\left(b^{2}/4\right)}}{a+c-\left(b/2\right)}$	$\sin^{-1}\left[\frac{a}{a+c}\cdot \sin\left(\varphi \text{SIGN}\varphi -\frac{\theta_{x}}{2}\right)\right]$	$\frac{a\cdot \sin\left(\varphi \text{SIGN}\varphi -\beta -\left(\theta_{x}/2\right)\right)}{\sin\beta }$
$\frac{\pi }{2}+\tan^{-1}\frac{\sqrt{a^{2}-\left(b^{2}/4\right)}}{a+c-\left(b/2\right)}<\left|\varphi \right|\leq \pi {‐\sin}^{-1}\frac{b}{2a}$	$\sin^{-1}\left[\frac{a}{a+c‐b}\cdot \sin\left(\varphi \text{SIGN}\varphi +\frac{\theta_{x}}{2}\right)\right]$	$\frac{a\cdot \sin\left(\varphi \text{SIGN}\varphi -\beta +\left(\theta_{x}/2\right)\right)}{\sin\beta }$
$\pi -\sin^{-1}\frac{b}{2a}<\left|\varphi \right|\leq \pi$	0	2*a* + *c* − *b*

## Data Availability

The data used to support the findings of this study are available from the corresponding author upon request.
